# Herbal or traditional medicine consumption in a Thai worker population: pattern of use and therapeutic control in chronic diseases

**DOI:** 10.1186/s12906-019-2652-z

**Published:** 2019-09-18

**Authors:** Napatt Kanjanahattakij, Pakakrong Kwankhao, Prin Vathesatogkit, Nisakron Thongmung, Yingampa Gleebbua, Piyamitr Sritara, Chagriya Kitiyakara

**Affiliations:** 1 Department of Medicine, Faculty of Medicine, Ramathibodi Hospital, Mahidol University, Bangkok, 10400 Thailand; 2 Department of Medicine, Einstein Medical Center, Philadelphia, PA 19141 USA; 3 Pharmacy department, Chao Phya Abhaibhubejhr hospital, Prachinburi, 25000 Thailand; 4 Research Center, Faculty of Medicine, Ramathibodi Hospital, Mahidol University, Bangkok, 10400 Thailand; 5 Medical and Health Division, Electricity Generating Authority of Thailand, EGAT 53 Moo 2 Charansanitwong Road,Bangkruai, Nonthaburi, 11130 Thailand

**Keywords:** Asia, Cardiovascular, Diabetes, Hypertension, Herbs, Non-communicable diseases, Thai, Traditional medicine Asia & Oceania

## Abstract

**Background:**

Herbal and traditional medicines (HTM) are widely used in Asian countries. Specific data on prevalent of HTM usage and association with chronic diseases in the Thai population is currently lacking. We examined the prevalence and factors associated with HTM use in a Thai worker population. In addition, we explored the relationship between HTM use and therapeutic control of cardiovascular risk factors and documented the most common types of HTM used in various chronic diseases.

**Methods:**

Employees of EGAT (The Electric Generating Authority of Thailand) who had participated in a health examination were studied. Each participant documented their HTM consumption and self-reported chronic diseases in a questionnaire. Clinical disease and therapeutic control were also defined by concomitant laboratory tests.

**Results:**

Of a total of 6592 subjects, 32.6% were HTM-users. Age < 50 years, female gender, self-reported history of diabetes, liver disease, cancer, dyslipidemia, and alcohol use were independently associated with HTM use. HTM consumption increased in proportion to the numbers of self-reported chronic diseases. There were no differences in the therapeutic control of cardiovascular risk factors between HTM users and non-users. Liver and kidney function were not different. The most commonly used HTM was turmeric.

**Conclusions:**

HTM consumption is common in community-based Thai subjects, with higher use among those with chronic diseases. Although there were no differences in control of cardiovascular risk factors between HTM users and non-users, many of the commonly used herbs have relevant biological activities for chronic disease prevention or treatment.

**Electronic supplementary material:**

The online version of this article (10.1186/s12906-019-2652-z) contains supplementary material, which is available to authorized users.

## Background

Rapid urbanization and globalization has led to a marked increase in non-communicable diseases (NCDs) around the world [1]. As the pathological processes linked to NCD may take years to develop, the use of preventive medicine to target risk factors during the pre-symptomatic period might prevent the development of cardiovascular disease (CVD) or other chronic diseases. Continuous management of subclinical pathology requires very safe agents to be regularly taken for an individual’s lifetime. The use of herbal medicine either for primary prevention or as complementary and alternative medicine for CVD risk factors such as hypertension or diabetes and other NCD is thus of increasing interest to the public and the medical community [2].

The World Health Organization (WHO) estimated that 70–80% of populations from developing countries use herbal and traditional medicine (HTM) as the primary method for health care needs, and HTM use has also been extensively embraced in Western countries [3]. Despite the widespread use for centuries, HTM is often approached with skepticism by the medical community [4] and evidence-based studies of the efficacy and safety of HTM in the management of chronic diseases are still limited [2]. At present, few large population surveys have examined HTM use in conjunction with laboratory and clinical data. The relationship between HTM consumption and the level of therapeutic control of CVD risk factors in the general population is still relatively unknown. HTM may improve the control of CVD risk factors either through direct pharmacological effects and HTM users might be more proactive to health risk modifications. On the other hand, HTM has also been associated with worsening kidney function [5] or liver toxicity [6].

In 2011, Thailand was reclassified by the World Bank from a lower-middle income to a higher-middle-income country. Along with the economic transition, the prevalence of CVD and related risk factors have increased markedly [7]. Self-prescribed herbal medicine is common among patients attending healthcare facilities in Southeast Asia, [8, 9] but there is limited information on the relationship of HTM usage with chronic diseases in the Thai community at large. The main aims of this study were to examine the prevalence and factors associated with HTM use in a Thai worker population. In addition, we will explore the relationship between HTM use and therapeutic control of CVD risk factors, and also document the most common types of HTM used in various chronic diseases.

## Methods

### Study subjects

The subjects were employees of EGAT (The Electric Generating Authority of Thailand), who had participated in a health survey to evaluate risk factors for cardiovascular and other chronic illnesses. The details of this cohort has been described in detail elsewhere [10]. This study is a part of the EGAT study’s cross-sectional survey from 2007 to 2009, in which 6796 employees or former employees of EGAT aged 25 to 76 agreed to participate. Every participant received a full medical history and physical examination by a trained medical personnel. A set of standardized, detailed questionnaires was also given to each participant to inquire about their demographic data and general health including questions about awareness of specific chronic diseases. (Additional file 1) In the questionnaire, the participants were asked if they used any HTM. Participants who responded “yes” were classified as a ‘HTM user’. Participants who responded “no” were classified as a ‘Non-user ‘. Participants who did not respond to this question were excluded from the study. HTM users were also asked to list the type of HTM they had used in the questionnaire. Blood samples were drawn after 12 h fast. This study was conducted in accordance with the Helsinki Declaration, and approved by the Ethics Committee, Ramathibodi Hospital, Mahidol University, Thailand (Protocol approval ID-05-51-19). Written informed consent was obtained.

### Clinical and laboratory disease definitions

Smokers and alcohol drinkers were defined according to status within 1 year prior to the survey. Obesity was defined as BMI > 30 kg/m^2^. Diabetes was defined as a fasting glucose of ≥126 mg/dl, or use of oral hypoglycemic medications or insulin or those with previous history of diabetes mellitus [11]. Hypertension was defined as a finding of average systolic BP ≥ 140 mmHg or diastolic BP ≥ 90 mmHg or use of anti-hypertensive medications or those with previous history of hypertension [12]. Chronic kidney disease (CKD) was defined as estimated glomerular filtration rate (eGFR) was less 60 ml/min/1.73m^2^ [13]. Severe CKD referred to those with eGFR< 45 [13]. Mild liver enzyme abnormality was defined as 1.5x upper limit of normal for alanine aminotransferase (1.5x ALT) or 1.5x aspartate aminotransferase (1.5 x AST). Severe liver enzyme abnormality was considered if either ALT or AST were × 3 above upper limit of normal.

### Self-reported disease definitions

Participants were said to have ‘self-reported’ diabetes, hypertension, liver disease, cancer, and dyslipidemia if they checked ‘Yes’ in specific boxes for the presence of these underlying conditions in the questionnaire. The disease was said to be absent if the subject checked ‘No’. Subjects who did not check the boxes were classified as missing. Previous cardiovascular disease was assigned if the patient had a history coronary heart disease or stroke. Each of the above self-reported condition was counted as one co-morbidity and the total number of self-reported co-morbid conditions were recorded for each individual.

### Laboratory measurements

Blood tests were performed in a laboratory in compliance with ISO 15189 as detailed previously [10]. Estimated Glomerular filtration rate (eGFR in ml/min/1.73m^2^) was calculated by using the CKD-EPI equation [13].

### Statistical analysis

Categorical data were reported as number (percent) and continuous data were reported in mean ± standard deviation. Demographic, clinical and laboratory data of HTM users were compared with Non-users by T test (continuous data) or Chi-square test (categorical). HTM use and the numbers of self-reported chronic conditions was evaluated by chi-square test. Factors associated with the use of HTM was evaluated by univariate and multivariate regression analysis using self-reported chronic conditions, CVD risk factors and demographic factors. The proportion within therapeutic targets for self-reported conditions were compared between HTM users and non-users. The top ten most common types of HTM used by self-reported diseases or laboratory-defined liver enzyme or kidney function abnormalities were identified. Statistical analysis was performed using SPSS version 22 (IBM, Armonk, NY) Missing data were excluded. Tests were 2-tailed, and a *p*-value < 0.05 was considered statistically significant.

## Results

### Demographic, clinical and laboratory characteristics of HTM users

**Fig. 1:**
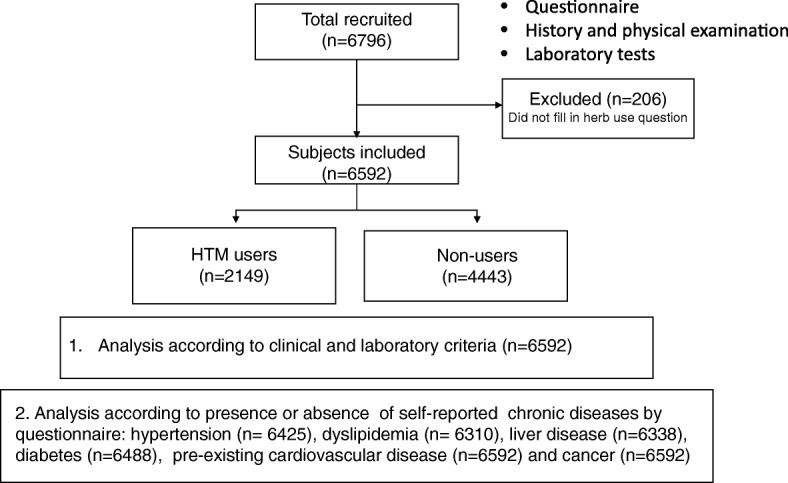
Flow of the study participants according to herb usage in the health questionnaire. HTM users, herb and traditional medication users

After exclusion of participants (*n* = 204) who did not respond to the HTM use question, a total of 6592 subjects (4810 males and 1782 females) or 97% of those originally recruited were included in the study (Fig. 1). Overall, age was 53 ± 11.3 years, 12.2% were diabetic and 40.2% were hypertensive.

The prevalence of HTM use was 32.6% (*n* = 2149). Characteristics of HTM users were compared with Non-users (Table 1). A higher proportion of females took HTM**.** Of interest, HTM users were more likely to consume alcohol within the last year. The prevalence of diabetes was higher among HTM users. No differences were observed in blood chemistry values. The eGFR, the proportion of CKD, and severe CKD (eGFR< 45) (*data not shown)* were similar between the 2 groups. Few subjects had severe liver dysfunction (ALT × 3 normal 1%, AST × 3 normal 0.3%). Proportions with mild or severe liver enzyme abnormality were similar between HTM users and Non-users.

**Table 1:** Demographic and laboratory characteristics according to HTM use

Variables	HTM usage		*p*-value
	HTM Users (*n* = 2149)	Non-Users (*n* = 4443)	
Male (%)	1522 (31.6)	3288 (68.3)	**0.007**
Female (%)	627 (35.2)	1155 (64.8)	
Age (years)	53 ± 11	53 ± 11	0.6
Education (%)			0.6
High school/ Bachelor/Master+	46/41/13	45/42/13	
Household Income (%)			0.4
< 20,000/20,000-49,999/50,000-100,000/> 100,000 THB/month	13/34/39/15	12/33/39/16	
Current/Never smoker (%)	16/45	17/44	0.5
Current Alcohol (%)	63	59	**0.003**
Body mass index (kg/m^2^)	25 ± 3.7	25 ± 3.6	0.3
Obese (%)	8	8	0.2
Systolic BP (mmHg)	126 ± 18	126 ± 18	0.3
Diastolic BP (mmHg)	81 ± 11	81 ± 11	0.5
Hypertension (%)	41	40	0.3
Fasting Glucose (mg/dl)	100 ± 31	99 ± 26	0.1
Diabetes Mellitus (%)	14	12	**0.006**
Triglyceride (mg/dl)	136 ± 87	140 ± 90	0.1
HDL (mg/dl)	53 ± 14	53 ± 14	0.4
LDL (mg/dl)	146 ± 40	146 ± 39	0.5
Creatinine (mg/dl)	1.02 ± 0.35	1.01 ± 32	0.7
eGFR (ml/min/1.73m^2^)	83 ± 20	83 ± 19	0.7
CKD (%)	12	11	0.1
1.5 x AST (%)	2	2	0.7
1.5 x ALT (%)	14%)	14%	0.8

Abbreviations: *BP* blood pressure, *HDL* high density lipoprotein, *LDL* low density lipoprotein, *eGFR* estimated glomerular filtration rate *CKD* chronic kidney disease

1.5x AST, 1.5 x upper limit of normal value for alanine aminotransferase

1.5x ALT1.5 x upper limit of normal value for aspartate aminotransferase

Bold *p* values  < 0.05

### HTM consumption and self-reported chronic diseases

Of all 6592 subjects included, the numbers of subjects who responded to questions on presence or absence of specific chronic diseases by checking a box on the questionnaire were: hypertension (*n* = 6425, 97%), dyslipidemia (*n* = 6310, 96%) liver disease (*n* = 6338, 96%), diabetes (*n* = 6488, 98%), pre-existing CVD (*n* = 6592, 100%) and cancer (n = 6592, 100%) (Fig. 1). The prevalence of self-reported diseases were: hypertension (27.1%), dyslipidemia (27.1%), liver disease (11.2%), diabetes mellitus (10.2%), pre-existing CVD (4.4%) and cancer (3.9%). HTM users were more likely (*p* < 0.05) to have self-reported diabetes mellitus (HTM users, 13% vs Non-users, 9%),dyslipidemia (HTM users, 48% vs Non-users, 41%), liver diseases (HTM users, 14% vs Non-users, 10%) and cancer (HTM users, 5% vs Non-users, 4%) and tended to have higher proportion of self-reported hypertension, but there were no differences in established CVD. (Additional file 3: Figure S1) The prevalence of HTM users increased with numbers of self-reported co-morbidities (*p* < 0.001) (Additional file 4: Figure S2).

The laboratory values and the proportion out of recommended target range of subjects with self-reported conditions are shown in Additional file 2: Table S1. Among subjects with self-reported CVD risk factors or liver disease, the laboratory levels or proportion within recommended target controls for each condition were similar between HTM users and non-users.

### Factors associated with HTM use

To evaluate the factors potentially influencing HTM use, demographic factors or self-reported chronic diseases were evaluated for associations with HTM consumption (Table 2) By univariate analysis, female gender, self-reported diabetes mellitus, liver disease, dyslipidemia, cancer, and alcohol consumption were associated with HTM intake. Age < 50 tended to be associated with HTM use (*p* = 0.07). By multivariate analysis, younger age (< 50), female, self-reported diabetes, liver disease, dyslipidemia, cancer and current alcohol consumption were associated with HTM consumption.

**Table 2:** Factors associated with Herbal and Traditional Medicine use

	Univariate			Multivariate		
Variable	OR	95% CI	*P*-value	OR	95% CI	*P*-value
Age (> 50 vs < 50 years)	0.91	0.81–1.01	0.07	0.82	0.72–0.93	**0.002**
Sex (F vs M)	1.17	1.05–1.32	**0.006**	1.34	1.17–1.54	**< 0.001**
Obesity (Y vs N)	1.11	0.92–1.34	0.2			
Education (Secondary vs higher)	1.00	0.91–1.11	0.9			
Income (< 20,000 THB vs higher)	1.07	0.91–1.25	0.4			
Current smoker (Y vs N)	0.96	0.84–1.10	0.5			
Current alcohol (Y vs N)	1.18	1.06–1.31	**0.003**	1.30	1.30–1.14	**< 0.001**
SR Diabetes mellitus (Y vs N)	1.43	1.21–1.69	**< 0.001**	1.37	1.14–1.66	**0.001**
SR Hypertension (Y vs N)	1.12	1.00–1.26	0.058			
SR Liver disease (Y vs N)	1.42	1.21–1.67	**< 0.001**	1.39	1.17–1.64	**< 0.001**
SR Dyslipidemia (Y vs N)	1.31	1.18–1.46	**< 0.001**	1.33	1.18–1.50	**< 0.001**
SR Cancer (Y vs N)	1.38	1.07–1.78	**0.01**	1.32	1.003–1.74	**0.047**
SR CVD (Y vs N)	0.96	0.74–1.24	0.7			

*OR* Odds ratio, *CI* confidence interval; Obesity; BMI > 30 kg/m^2^; Income, Monthly household income in Thai Baht; *SR* self-reported, *CVD* cardiovascular disease

*p* < 0.05 denotes statistical significance

Bold *p* values  < 0.05

### Types of HTMs used in different diseases

One thousand two hundred subjects listed HTMs that they had used. Of these subjects, 208 subjects listed more than 1 compound. The top ten most commonly used HTM (in order from the most common first) were: turmeric (20%), cinnamon (19%), heart-leaved moonseed (18%), garlic (17%), mixed botanical preparations (13%), ginseng (13%), lingzhi mushroom (12%), kariyat (11%), black ginger (11%), and drumstick tree (10%).

The top 10 most commonly used HTMs in subjects with pre-existing self-reported conditions, or laboratory defined liver enzyme abnormality or CKD are shown in Table 3. Kariyat was the only HTM identified in participants with severe liver dysfunction (AST or ALT> 120). The list of drugs used in subjects with severe CKD (eGFR< 45) *(data not shown)* were similar to CKD (eGFR< 60).

**Table 3:** Top ten herbal and traditional medicine according to self-reported conditions or laboratory defined diseases

Self-reported disease					Laboratory-defined disease	
Diabetes	Hypertension	Dyslipidemia	Liver disease	Cancer	ALT × 1.5	CKD
Cinnamon (*Cinnamomum* spp.) Heart-leaved moonseed (*Tinospora crispa* (L.) Miers ex Hook.f. & Thomson), Safflowe*r* (*Carthamus tinctorius* Linn.) Black ginger (*Kaempferia parviflora* Wallich. ex Baker.) Lingzhi mushroom (*Ganoderma lucidum* (Curtis) P. Kars) Garlic (*Allium sativum* L.) Drumstick tree (*Moringa oleifera* Lam.) Turmeric (*Curcuma longa* L.) Mixed botanical preparations Ginseng (*Panax ginseng* C.A.Mey.)	Garlic (*Allium sativum* L.) Heart-leaved moonseed (*Tinospora crispa* (L.) Miers ex Hook.f. & Thomson), Cinnamon (*Cinnamomum* spp.) Lingzhi mushroom (*Ganoderma lucidum* (Curtis) P. Kars) Mixed botanical preparations Kariyat (*Andropraphis paniculata* (Burm.f.) Wall. Ex Nees) Drumstick tree (*Moringa oleifera* Lam.) Turmeric (*Curcuma longa* L.) Ginseng (*Panax ginseng* C.A.Mey.) Traditional alcohol with herbs	Heart-leaved moonseed (*Tinospora crispa* (L.) Miers ex Hook.f. & Thomson) Black ginger (*Kaempferia parviflora* Wallich. ex Baker.) Safflower (*Carthamus tinctorius* Linn.) Cinnamon (*Cinnamomum spp*.) Turmeric (*Curcuma longa L*.) Lingzhi mushroom (*Ganoderma lucidum* (Curtis) P. Kars) Kariyat (*Andropraphis paniculata* (Burm.f.) Wall. Ex Nees) Mixed botanical preparations Ginseng (*Panax ginseng* C.A.Mey.) Drumstick tree (*Moringa oleifera* Lam.)	Heart-leaved moonseed (*Tinospora crispa* (L.) Miers ex Hook.f. & Thomson) Mixed botanical preparations Cinnamon (*Cinnamomum spp*.) Lingzhi mushroom (*Ganoderma lucidum* (Curtis) P. Kars) Turmeric (*Curcuma longa* L.) Garlic (*Allium sativum* L.) Kariyat (*Andropraphis paniculata* (Burm.f.) Wall. Ex Nees) Ginseng (*Panax ginseng* C.A.Mey.) Drumstick tree (*Moringa oleifera* Lam.) Black ginger (*Kaempferia parviflora* Wallich. ex Baker.)	Lingzhi mushroom (*Ganoderma lucidum* (Curtis) P. Kars) Ginseng (*Panax ginseng* C.A.Mey.) Cinnamon (*Cinnamomum spp*.), Turmeric (*Curcuma longa* L.) Safflower (*Carthamus tinctorius* Linn.) Mixed botanical preparations Black ginger (*Kaempferia parviflora* Wallich. ex Baker.) Kariyat (*Andropraphis paniculata* (Burm.f.) Wall. Ex Nees)	Mixed botanical preparations Safflower (*Carthamus tinctorius* Linn.) Garlic (*Allium sativum* L.), Cinnamon (*Cinnamomum spp*.) Ginseng (*Panax ginseng* C.A.Mey.) Kariyat (*Andropraphis paniculata* (Burm.f.) Wall. Ex Nees) Heart-leaved moonseed (*Tinospora crispa* (L.) Miers ex Hook.f. & Thomson Lingzhi mushroom (*Ganoderma lucidum* (Curtis) P. Kars) Drumstick tree (*Moringa oleifera* Lam.) Turmeric (*Curcuma longa* L.) Black ginger (*Kaempferia parviflora* Wallich. ex Baker.)	Lingzhi mushroom (*Ganoderma lucidum* (Curtis) P. Kars) Black ginger (*Kaempferia parviflora* Wallich. ex Baker.) Ginseng (*Panax ginseng* C.A.Mey.) Turmeric (*Curcuma longa* L.) Cinnamon (*Cinnamomum* spp.) Garlic (*Allium sativum* L.) Kariyat (*Andropraphis paniculata* (Burm.f.) Wall. Ex Nees) Mixed botanical preparations Drumstick tree (*Moringa oleifera* Lam.)

Abbreviations: ALT × 1.5; 1.5 times upper limit of normal for alanine aminotransferase; CKD, chronic kidney disease defined as estimated glomerular filtration rate < 60 ml/min/1.73m^2^

## Discussion

In this study, we found that 33% of a Thai worker population used HTM. Age < 50 years, female gender, self-reported history of diabetes, liver disease, cancer, dyslipidemia, and alcohol use were independently associated with HTM use. HTM consumption increased in proportion to the numbers of self-reported chronic disease factors. There were no differences in the therapeutic control of CV risk factors between HTM users and non-users. The most commonly used HTM was turmeric.

The reported prevalence of traditional medicine users in Southeast Asia varied widely from 55% in Singapore to 2% in Indonesia [9]. In Thailand, data from National Health Survey in 2014 showed that 21.9% of Thai population had used HTM [14]. On the other hand, smaller surveys involving a few hundred subjects showed that 29 to 54% were using HTM [15, 16]. In our study of over 6000 subjects, we found a high percentage of HTM usage in a Thai worker population (33%) similar to previous smaller local surveys. The differences between various studies may be partially explained by differences in the reference period (ever used versus past year use) and inclusion of other health practices such as Thai massage, and meditation in the survey.

Chronic illnesses has been associated with higher HTM use in Western populations [17]. Surveys from Thai outpatient clinics showed that between 35 and 60% of patients with chronic diseases used HTM [8–19]. Our study of community -based workers demonstrated that chronic disease or known CVD risk factors appear to be associated with HTM use in a Thai general population, although HTM use was still quite high (30%) even in those without known pre-existing conditions. Obesity is also associated with higher HTM use in our study. Obesity is often associated with multiple risk factors for CVD and predisposes to many chronic diseases. Given high toxicities or lack efficacy of Western medications on the treatment of obesity, there has been considerable interests in the use of HTM to assist weight loss or prevent long term consequences of obesity [20]. Our study also showed that current alcohol intake was associated with HTM use. This is similar to results from the US, where drinkers were more likely to have used HTM, compared to lifetime teetotalers [21]. The relationship between alcohol and HTM use is complex, as many factors in life could influence both the use of HTM and alcohol consumption such as partner strain, pain and mental disorders such as major depression and panic disorders.

Although HTM are widely used in patients with established CVD, [22] the relationship of HTM use with CV risk factor control in the general population is unknown. We did not observe any differences in BP level or laboratory parameters between HTM users and non-users. Moreover, in subjects with known self-reported conditions, HTM use was not associated with better therapeutic control. These findings do not exclude potential benefits of specific HTM on CVD risk factors. The pharmacologic impact of specific HTMs might not be apparent due to the heterogeneity in preparations used in our study. Several factors may also affect control of CV risk factors including the pre-existing severity of the risk factors prior to HTM use which are not known. In addition, there is some evidence to suggest that rather than leading to an improved life-style, some HTM users may paradoxically have reduced compliance of prescribed medications because of a false sense of security of therapeutic control [22].

Most of the HTMs in our top ten list has been shown to have potential health benefits. In Thailand, turmeric is a commonly used culinary compound, listed in Thai National List of Essential Medicines as anti- flatulence agent [23]. Turmeric contains a variety of curcumins which have been shown in experimental studies to have lipid lowering, anti-oxidant, anticancer properties associated with minimum toxicity [24, 25]. Cinnamon was the most common HTM in subjects with self-reported diabetes. Several studies have shown that cinnamon may improve glycemic control in diabetics [26]. Of interest, some types of cinnamon contain coumarin, which may interfere with concomitant use of anticoagulants. Garlic is the most commonly used drug in subjects with self-reported hypertension. Garlic may help to reduce blood pressure, cholesterol, and inhibit platelet aggregation, although different garlic preparations may have variable effectiveness on blood pressure [27, 28]. Lingzhi mushroom possess immunomodulation and antioxidant properties as well as inhibitory effects on angiotensin converting enzyme [29]. These properties may account for their popularity in subjects with CKD [18] and cancer. The benefits of Lingzhi mushroom on quality of life has been shown in cancer patients [30]. Kariyat was used in subjects with self-reported liver disease and those with severe liver enzyme abnormality. Protective effects of Kariyat on liver disease has been shown in vitro and in vivo [31]. Of interest, Kariyat is used by Khmer traditional healers in Cambodia for treatment of liver disease perhaps reflecting common regional traditional practices [32]. Kariyat is also widely promoted for use as a therapy of common cold and diarrhea [33] which may account for its common usage in Thailand [34]. Though considered safe, serious allergic effects to Kariyat have been reported [35].

For some commonly used HTM, more studies are necessary to demonstrate potential health benefits. Black ginger (*Kaempferia parviflora*) has been used in Southeast Asia for centuries to improve physical work capacity and as an aphrodisiac. It has been selected as a champion herbal product to generate income in Thailand. A recent systematic review, however, failed to find conclusive evidence to support the benefits of *Kaempferia parviflora* [36]. Leaves, pod, seeds and oils from drumstick tree (*Moringa oleifera*) possess pharmacological activity based on pre-clinical studies in chronic diseases such as dyslipidemia, diabetes and hypertension [37]. Although the use of drum stick tree has recently been popularized in Thailand, there has been no comprehensive study of their pharmacological effects in humans [37, 38].

In Thailand, HTM is available mostly as traditional drugs or formulations, modified traditional drugs in modern dosage forms, e.g. capsules or tablets or as phytopharmaceuticals, which are composed of standardized active plant materials in the form of semi-purified compounds. In practice, the consumers do not differentiate between the different types of preparations or dosage and culinary herbs taken in higher quantities are often included as HTM. Mixed botanical preparations was listed by many HTM users. Typically, these preparations contain a wide-spectrum of herbs and the contents are not known by the consumers as the labelling of all ingredients are not legally enforced. Indeed, the majority of users were not able to list the HTM, they consumed. Although we did not determine the sources of HTM in this study, other studies in diabetic [19] and CKD patients [18] showed that although a proportion of HTM are prescribed by licensed traditional medicine practitioners, most HTM use in Thailand is self-prescribed with folk remedy shops, direct sale, markets or family being the commonest sources. Under these circumstances which may be unregulated, the subjects often do not know the types of HTM they consumed.

HTM especially the use of aristolochic acid in Chinese traditional medicine use has been shown to be associated with CKD [5]. Less frequent use of aristolochic acid might account for the lack of association of HTM use with CKD in our subjects. Overall, we did not demonstrate any association between abnormal liver function tests and HTM consumption. However, heart-leaved moonseed (believed to have important immune-modulation properties was among the most commonly used HTM in those with mildly elevated liver function tests. This drug has been reported to cause toxic hepatitis [39]. Thus, vigilance may be necessary with its use.

This study has several implications. First, the high prevalence of HTM use in the community should raise the vigilance of the Thai healthcare professionals to inquire about HTM use in their patients. Medical practitioners are often unaware of HTM use, either due to the patients’ reluctance to disclose HTM use, or because the physicians do not ask [19]. HTM may interact with prescribed medications leading to loss of efficacy or increased adverse effects. The likelihood of herb-drug interactions may be higher in the 20% of subjects who take more than one type of HTM [22]. In addition, compliance of prescribed drugs need to be specifically addressed among HTM users. Secondly, there is a special concern regarding the lack of knowledge regarding the types of HTM consumed. Regulatory requirements to certify HTM preparations and control the distribution of products with unknown biological effects are necessary to minimize potential harm [3]. Meanwhile, patients should be educated to observe the safety and efficacy of OTC HTMs such as turmeric, kariyat by their own as distribution channel of OTC as HTM quality may not be regulated by health professionals. Finally, many of the commonly used HTM possess pharmacologic effects which should be explored further in the context of clinical trials and the long term side-effects recorded in registries. All these issues are especially important now as the Thai Government has new policies to develop of the Thai HTM industry as a key economic driver, which will likely increase the use of HTM.

The strengths of our study are that this is one of the largest study on HTM consumption in Southeast Asia and one of the few studies to evaluate the relationship between HTM consumption with chronic diseases and CV risk factor control using both clinical and laboratory parameters in community-based subjects. Unlike most previous studies, which have generally been in the form of questionnaires, our data was obtained by trained medical personnel with special emphasis on chronic disease detection employing standardized laboratory procedures. There are several limitations of the study. This was a cross-sectional analysis, and causal relationship between HTM use and disease status or therapeutic control cannot be established. Data was not available on the dose or duration of HTM use, which would be important in defining HTM exposure. In addition, we did not have data on the reason or expectations of subjects for using HTM. Therefore, our study probably reflects the HTM prevalence and consumption behavior rather than the effects of specific HTM on CVD control. The list of HTM was by obtained by recall and many people did not list the HTM used. Thus the data represented a general pattern of HTM prevalence rather than a formal assessment of specific HTM use. Nonetheless, the common HTM identified in this community-based study are similar to other studies from Thai outpatient clinics [18, 19]. Finally, while our findings should be generally applicable to the Thai community, our studied subjects had a higher proportion of males and consisted of a worker population that may differ slightly from the Thai population as a whole. Although EGAT employees come from all regions of Thailand and cover a wide-range of demographic backgrounds, the socio-economic status of EGAT employees is probably better than some of the most severely economically disadvantaged Thais, and the study did not include the severely ill or disabled subjects excluded from employment [10]. Nonetheless, the prevalence of CVD risk factors found in our study subjects are comparable to those found in nationally representative surveys [40].

## Conclusions

HTM consumption is common in community-based Thai subjects, with higher use among those with chronic diseases. Although there were no differences in control of CVD risk factors between HTM users and non-users, many of the commonly used herbs have relevant biological activities for prevention or treatment of chronic diseases. Health care professionals should ask specifically about HTM use to minimize interactions between HTM and prescribed drug. Further studies on the specific HTM should be conducted to demonstrate the risks and benefits for chronic diseases management. Lastly, health authorities in Thailand should promote the certification of HTM, provide regulations for HTM sale, and evidence-based guidelines for HTM use as a part of standard practice.

## Additional files

Additional file 1: EGAT questionnaire (DOCX 21 kb)

Additional file 2: **Table S1.** The laboratory values and the proportion out of recommended target range of subjects with self-reported conditions. (DOCX 17 kb)

Additional file 3: **Figure S1.** Prevalence of self-reported conditions among herb and traditional medication (HTM) users compared to non-users. Shaded bars reprsent HTM users; white bars represent Non-users. * denotes *p* < 0.05. (PDF 8 kb)

Additional file 4: **Figure S2.** Prevalence of herb and traditional medication users by numbers of self-reported non-communicable disease conditions. (PDF 32 kb)

## Data Availability

The datasets used and/or analyzed during the current study are available from the corresponding author on reasonable request.

## Abbreviations

ALT: aspartate aminotransferase
AST: alanine aminotransferase
BP: blood pressure
CKD: chronic kidney disease
CVD: cardiovascular disease
DM: diabetes mellitus
EGAT: Electric Generating Authority of Thailand
eGFR: estimated glomerular filtration rate
HDL: high density lipoprotein
HTM: herb and traditional medicaqtions
LDL: low density lipoprotein
NCD: non-communicable diseases
OTC: over the counter
