# Supplementary material for: Herbal or traditional medicine consumption in a Thai worker population: pattern of use and therapeutic control in chronic diseases
Source: BMC Complement Altern Med. 2019 Sep 18;19:258. doi: 10.1186/s12906-019-2652-z (PMC6749623; doi:10.1186/s12906-019-2652-z)
Supplement: Supplementary file 1 — EGAT questionnaire (DOCX 21 kb) [file 12906_2019_2652_MOESM1_ESM.docx]

**EGAT Questionnaire v 2007-2009 (translated from Thai version)**

**1. Subject details**

First Name……………………………… Last name……….……………………

Address……………………………………………………………………………………………

Telephone number: Home / mobile 1.……………2……………Office……………………

National ID |__|__|__|__|__|__|__|__|__|__|__|__|__|

**2. Contact relative**

First Name……………………………… Last name……….……………………

Address……………………………………………………………………………………………

Telephone number: Home / mobile 1.……………2……………Office……………………

**3. Working status**

_ 1. Work at EGAT _ 2. Retired _ 3. Work elsewhere

**4. Date of birth**

|__|__| |__|__| |__|__|__|__| or Age |__|__|__| years

Day month Year

**5. Marital status**

_ 1. Single _ 2. Married

_ 3. Widowed _4. Divorced

**6. Education**

_ 1. Secondary or lower _ 2. Vocational _ 3. Bachelor _ 4. Master

_ 5. PhD _ 9. Don’t know

**7. Family income (baht per month)**

_ 1. Less than 10,000 _ 2. 10,000 – 19,999

_ 3. 20,000 – 49,999 _ 4. 50,000 – 99,999

_ 5. Above 100,000 _ 9. ไม่ทราบ

Numbers of people dependent on this income |__|__| people

**History of illnesses**

**8. Have you ever had the following illnesses**

1. Diabetes

**_** 1. No

_ 2. Yes

_ 9. Don’t know

2. Hypertension

_ 1. No

_ 2. Yes

_ 9. Don’t know

3. Dyslipidemia

_ 1. No

_ 2. Yes

_ 9. Don’t know

4. Cardiovascular disease

i) *Stroke*

_ 1. No

_ 2. Yes

_ 9. Don’t know

ii) *Coronary artery disease*

_ 1. No

_ 2. Yes

_ 9. Don’t know

iii) *Myocardial infarction*

_ 1. No

_ 2. Yes

_ 9. Don’t know

iv) *Heart failure*

_ 1. No

_ 2. Yes

_ 9. Don’t know

*v) Peripheral vascular disease*

_ 1. No

_ 2. Yes

_ 9. Don’t know

*vi) Coronary angioplasty*

_ 1. No

_ 2. Yes Date

_ 9. Don’t know

*vii) Coronary artery bypass graft surgery*

_ 1. No

_ 2. Yes Date

_ 9. Don’t know

5. Liver disease

_ 1. No

_ 2. Yes

_ 9. Don’t know

6. Cancer

_1. No

_ 2. Yes

_ 9. Don’t know

If yes which type_________

Diagnosed at which hospital _______, date_______

Treatment 🞏 surgery 🞏chemotherapy 🞏 radiation

**9. List of medications**

_ 01 Aspirin ……………………………..............................…

_ 02 Acetaminophen………………………............…………..

_ 03 ACE – I …………………………..................……………

_ 04 Beta – blocker ………………….....................………….

_ 05 Calcium channel blocker …………..............................

_ 06 Cholesterol lowering drug ………….............................

_ 07 Digoxin………………………..................….…...............

_ 08 Diuretics………………….................….………..............

_ 09 Nitrates………………………........................…….. ...…

Diabetic meds

_ 10 Insulin ……………………….…..…….................…….…

_ 11 Oral hypoglycemic drug….………….................………

Hormones

_ 12 Hormone…..…………………………..…....................…

_ 13 Contraceptive pills…………………..……...................

_ 14 Contraceptive subcutaneous infiltration….................

Other medications

.................................................................................................

.................................................................................................

.................................................................................................

**10. Have you ever smoked cigarettes (>100 cigarettes or 5 packs lifetime)**

_ 1. No (If no go to #11)

_ 2. Yes

_ 9. Don’t know

1. At what age did you start smoking regularly?

🞏🞏 Years

_ Never regular

_ 9 Don’t know

1. Are you smoking now or not?

_1.No

_2.Yes

_9. Don’t know

1. On average how many cigarettes do you smoke a day?

🞏🞏 /day

_ <1 cig per day

_ 9 Don’t know

1. How long have you smoked that amount?

🞏🞏 years

_ 0-1 year

_ 9. Don’t know

1. Have you used other tobacco?

_1. No

_2. Pipe

_3. Cigar

_4.Chewing tobacco

_9. Don’t know

**11. Did you drink alcohol**

_1.Never

_2.Very little -less than once a month

_3.Used to drink regularly but stopped 🞏🞏 year

_4.Still drink for 🞏🞏 years

_9. Don’t know

**12 Did you ever use herbal and traditional medicines**

_ 1. No

_ 2. Yes

If yes, please list.

1...............................................................................................

2...............................................................................................

3...............................................................................................

4...............................................................................................

5...............................................................................................
