# Supplementary material for: Herbal or traditional medicine consumption in a Thai worker population: pattern of use and therapeutic control in chronic diseases
Source: BMC Complement Altern Med. 2019 Sep 18;19:258. doi: 10.1186/s12906-019-2652-z (PMC6749623; doi:10.1186/s12906-019-2652-z)
Supplement: Supplementary file 2 — Table S1. The laboratory values and the proportion out of recommended target range of subjects with self-reported conditions. (DOCX 17 kb) [file 12906_2019_2652_MOESM2_ESM.docx]

**Supplementary Table S1: Clinical and laboratory parameters in self-reported chronic diseases according to HTM use.**

| **Variables** | **HTM user** | **Non-User** | **P-value** |
| --- | --- | --- | --- |
|  | | | |
| Self-reported Diabetes | n = 262 | n = 384 |  |
| Fasting glucose, mg/dl | 148 ±59 | 143 ±48 | 0.20 |
| Fasting glucose >130 mg/dl, No (%) | 139 (53%) | 183 (48%) | 0.33 |
|  | | | |
| Self-reported hypertension | n = 589 | n =1101 |  |
| Systolic blood pressure, mmHg | 139±20 | 139±17 | 0.75 |
| Diastolic blood pressure, mmHg | 86±11 | 86±11 | 0.51 |
| Systolic blood pressure >140, No (%) | 230 (39%) | 457 (42%) | 0.35 |
| Diastolic blood pressure >90, No (%) | 211 (36%) | 376 (34%) | 0.52 |
| Severe hypertension (SBP >160 mm Hg), No (%) | 71 (12%) | 133 (12%) | 1.0 |
|  | | | |
| Self-reported dyslipidemia | n = 951 | n = 1696 |  |
| LDL, mg/dl | 150±43 | 150±43 | 0.97 |
| HDL, mg/dl | 54±14 | 53±14 | 0.37 |
| LDL>130 mg/dl. , No (%) | 636 (67%) | 1118 (66%) | 0.64 |
| HDL<40 mg/dl, No (%) | 813 (86%) | 1434 (85%) | 0.53 |
| Triglyceride>150 mg/dl, No (%) | 394 (41%) | 704 (42%) | 1.00 |
|  | | | |
| Self-reported liver disease | n = 276 | n = 425 |  |
| 1.5 x AST, , No (%) | 13 (42%) | 18 (58%) | 0.85 |
| 3 x AST, No (%) | 4 (1%) | 5 (1%) | 0.74 |
| 1.5x ALT, No (%) | 75 (40%) | 114 (60%) | 0.93 |
| 3x ALT, No (%) | 8 (3%) | 15 (4%) | 0.83 |

Data shown as mean ± SD or number (%).

Abbreviations: BP, blood pressure; HDL, high density lipoprotein; LDL, low density

lipoprotein; AST, alanine aminotransferase; ALT, aspartate aminotransferase
