# Supplementary figures and images for: Herbal or traditional medicine consumption in a Thai worker population: pattern of use and therapeutic control in chronic diseases
Source: BMC Complement Altern Med. 2019 Sep 18;19:258. doi: 10.1186/s12906-019-2652-z (PMC6749623; doi:10.1186/s12906-019-2652-z)

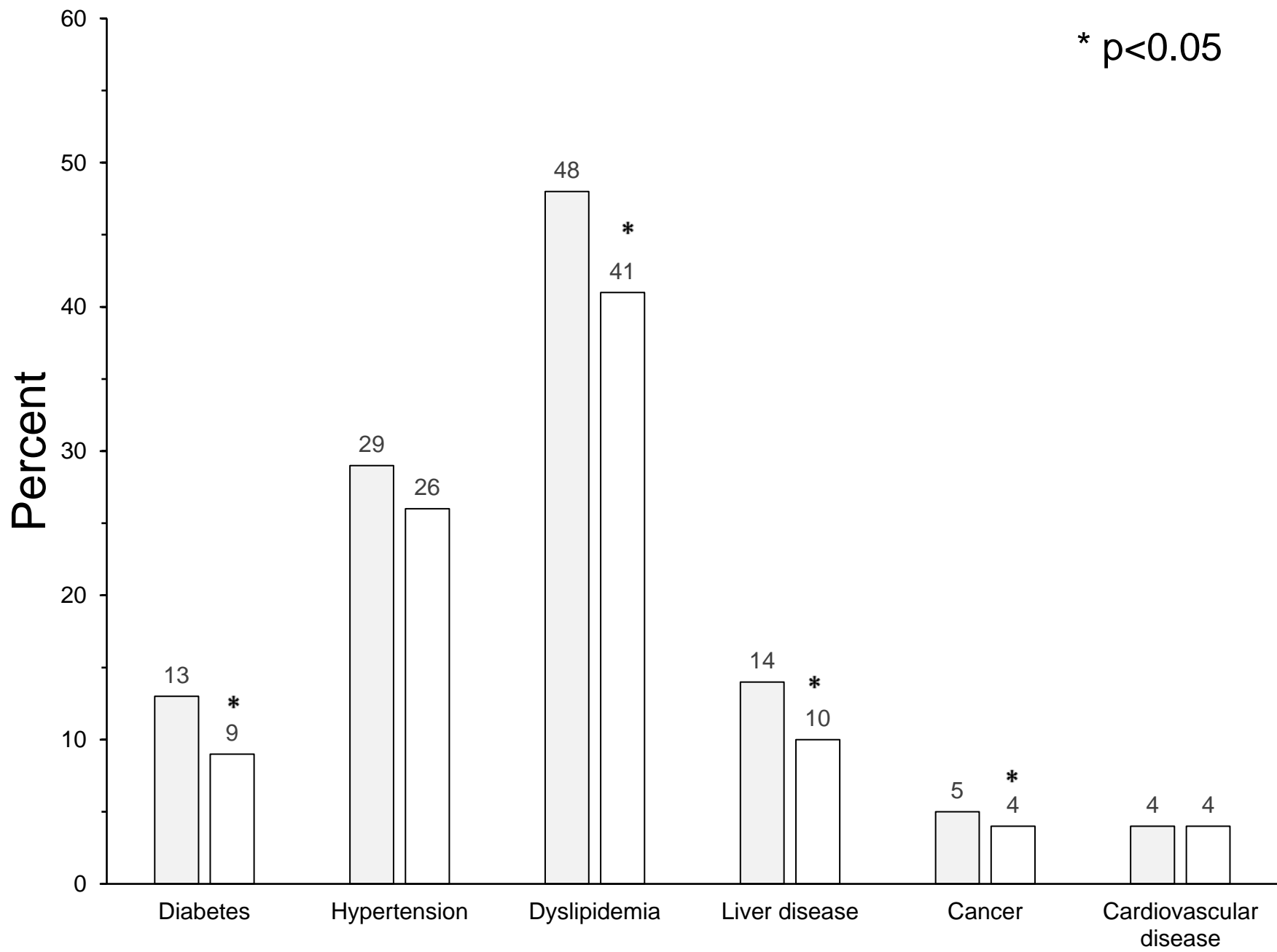

Supplement: Supplementary file 3 — Figure S1. Prevalence of self-reported conditions among herb and traditional medication (HTM) users compared to non-users. Shaded bars reprsent HTM users; white bars represent Non-users. * denotes p < 0.05. (PDF 8 kb) [file 12906_2019_2652_MOESM3_ESM.pdf]

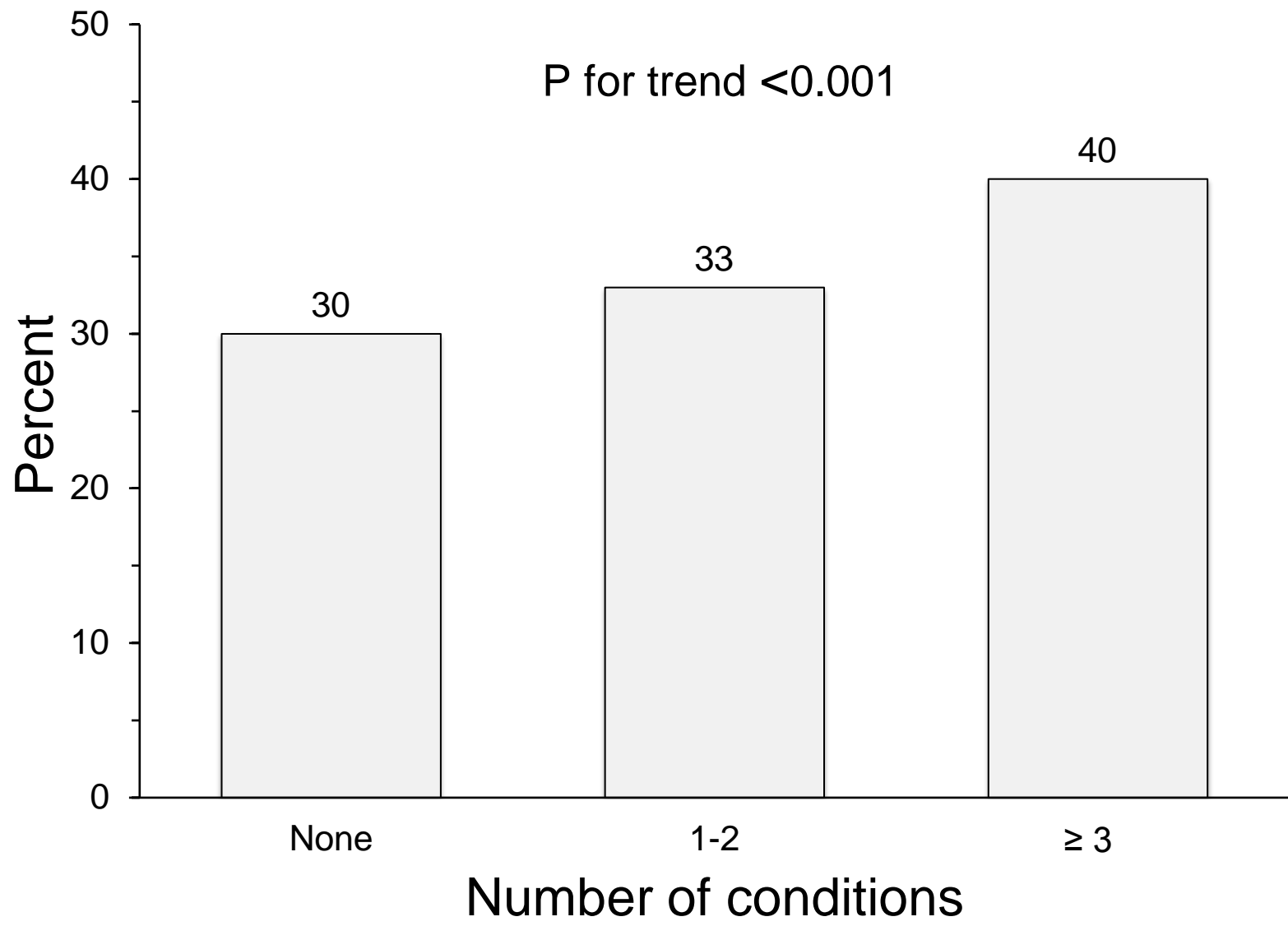

Supplement: Supplementary file 4 — Figure S2. Prevalence of herb and traditional medication users by numbers of self-reported non-communicable disease conditions. (PDF 32 kb) [file 12906_2019_2652_MOESM4_ESM.pdf]
